# Potential allopolyploid origin of Ericales revealed with gene-tree reconciliation

**DOI:** 10.3389/fpls.2022.1006904

**Published:** 2022-11-15

**Authors:** Shuai Nie, Xue-Chan Tian, Lei Kong, Shi-Wei Zhao, Zhao-Yang Chen, Si-Qian Jiao, Yousry A. El-Kassaby, Ilga Porth, Fu-Sheng Yang, Wei Zhao, Jian-Feng Mao

**Affiliations:** ^1^ Beijing Advanced Innovation Center for Tree Breeding by Molecular Design, National Engineering Laboratory for Tree Breeding, Key Laboratory of Genetics and Breeding in Forest Trees and Ornamental Plants, Ministry of Education, College of Biological Sciences and Technology, Beijing Forestry University, Beijing, China; ^2^ Henan Key Laboratory of Germplasm Innovation and Utilization of Eco-economic Woody Plant, Pingdingshan University, Pingdingshan, China; ^3^ Department of Forest and Conservation Sciences, Faculty of Forestry, University of British Columbia, Vancouver, BC, Canada; ^4^ Départment des Sciences du Bois et de la Forêt, Faculté de Foresterie, de Géographie et Géomatique, Université Laval, Québec, QC, Canada; ^5^ State Key Laboratory of Systematic and Evolutionary Botany, Institute of Botany, Chinese Academy of Sciences, Beijing, China; ^6^ China National Botanical Garden, Beijing, China; ^7^ University of Chinese Academy of Sciences, Beijing, China; ^8^ Department of Ecology and Environmental Science, Umeå Plant Science Centre, Umeå University, Umeå, Sweden

**Keywords:** Ericales, allopolyploidization, whole genome duplication, gene loss, hybridization

## Abstract

Few incidents of ancient allopolyploidization (polyploidization by hybridization or merging diverged genomes) were previously revealed, although there is significant evidence for the accumulation of whole genome duplications (WGD) in plants. Here, we focused on Ericales, one of the largest and most diverse angiosperm orders with significant ornamental and economic value. Through integrating 24 high-quality whole genome data selected from ~ 200 Superasterids genomes/species and an algorithm of topology-based gene-tree reconciliation, we explored the evolutionary history of in Ericales with ancient complex. We unraveled the allopolyploid origin of Ericales and detected extensive lineage-specific gene loss following the polyploidization. Our study provided a new hypothesis regarding the origin of Ericales and revealed an instructive perspective of gene loss as a pervasive source of genetic variation and adaptive phenotypic diversity in Ericales.

## Introduction

**Figure 1:**
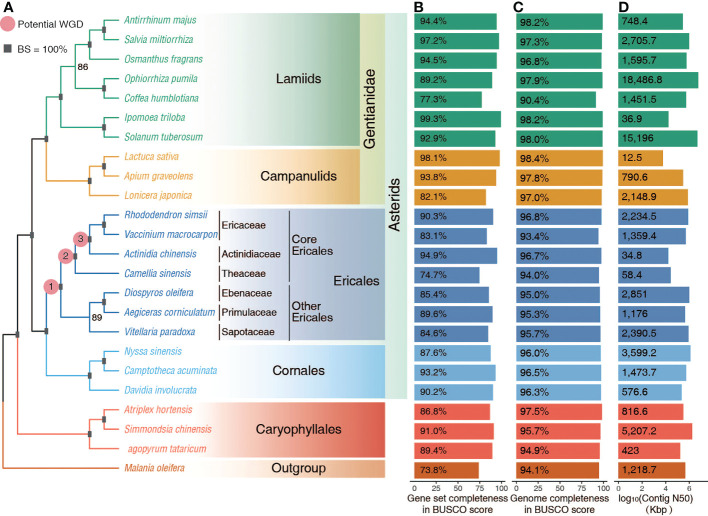
Whole genome data collected. **(A)** The inferred ML phylogeny of representative Superasterids based on the concatenated alignment of amino acid sequences of 532 orthogroups. Squares represent 100% bootstrap support (BS) values for nodes. The three pink dots represent the three possible WGD events identified in previous reports. **(B, C)** Completeness of genome assembly and gene annotation as reflected in Benchmarking Universal Single Copy Orthologs (BUSCO) scores. **(D)** Contig N50 of whole genome assemblies.

Ericales, with 22 families (~12,000 species), is a diverse and large clade of significant ornamental and economic value (APG IV, 2016) and provides an ideal system to investigate the mechanisms underlying the complex evolutionary history of flowering plants (Rose et al., 2018; Larson et al., 2020). It is quite possible that Ericales have experienced one ancient whole genome duplication (WGD) associated with global climate change after the gamma triplication of core eudicots. However, this WGD event has been inferred at different nodes at the common ancestors of: 1) the Ericales (Landis et al., 2018; Larson et al., 2020; Stull et al., 2020; Wang et al., 2021); 2) the core Ericales (Zhang et al., 2020b); and 3) the Ericaceae and Actinidiaceae (Yang et al., 2020) (**Figure 1A**). Furthermore, a phylogenetic network analysis inferred Ericales to be a reticulate lineage and have experienced incomplete lineage sorting as a result of rapid radiation occurred along the backbone of Ericales (Stull et al., 2020), thus an allopolyploid origin of this order could be expected. Differential gene loss and retention following WGD may greatly complicate our ability to resolve the origin and phylogenetic position of Ericales (Larson et al., 2020; Stull et al., 2020; Zhang et al., 2020b), and identifying this ancient WGD presents a key step in phylogenetic reconstruction of this species-rich plant order.

WGD, or polyploidization, is one of the major evolutionary processes that shape eukaryotic genomes, being particularly common in plants (Fawcett et al., 2009; Soltis et al., 2009; Jiao et al., 2011). WGD is considered an effective mechanism for surviving drastic climate changes (Fawcett et al., 2009; Zhang et al., 2020c). Polyploids can arise through direct genome doubling within a species (autopolyploidization) or through the merging of genomes of distinct species after hybridization (allopolyploidization) (Soltis et al., 2007; Hegarty and Hiscock, 2009). Compared to autopolyploidization, allopolyploidization provides more genomic opportunities and enhanced genomic plasticity for contributing to genetic diversification, speciation, and crop domestication. Due to the lack of polysomic inheritance and higher fertility rates, allopolyploids are more prevalent than autopolyploids within angiosperms, as it is reflected in well-characterized allopolyploids such as wheat (Feldman and Levy, 2005), cotton (Chen et al., 2007), and oilseed rape (Chalhoub et al., 2014).

Many methods have been employed to study and identify polyploidy events, including non-phylogenetic methods (such as synteny and karyotyping) (Barker et al., 2016; Kellogg, 2016), and phylogenetic methods (Ks-based, least common ancestor (LCA) reconciliation, gene networks, and count-based) (Page, 1994; Lynch and Conery, 2000; Linder and Rieseberg, 2004; Rabier et al., 2014). Despite the fact that most of these detection methods were used (Shi et al., 2010; Landis et al., 2018; Wei et al., 2018; Chen et al., 2020; Larson et al., 2020; Stull et al., 2020; Yang et al., 2020; Zhang et al., 2020b; Wang et al., 2021), the placement of the ancient WGD of Ericales has not been consistently resolved. These commonly used methods can correctly resolve cases of recent autopolyploidy and allopolyploids, yet they cannot accurately identify ancient allopolyploids as it is difficult to distinguish paralogs from orthologs of two parental species (Hegarty and Hiscock, 2009; Edger et al., 2018). For example, Ks- and LCA-based methods usually cannot distinguish the time of allopolyploidization and the divergence time of its two parental species (Gregg et al., 2017). Species networks can correctly represent species-level relationships for allopolyploids, but may be less practical for analyses involving multiple individual genes in allopolyploid genomes (Gregg et al., 2017). An alternative and more suitable LCA algorithm was developed for use with multi-labelled (MUL) trees to detect allopolyploidization, which has been implemented in the software package Gene-tree Reconciliation Algorithm with MUL trees for Polyploid Analysis (GRAMPA) (Gregg et al., 2017). By matching gene trees with species trees represented as MUL trees, conclusions can be drawn about the occurrence and nature of polyploidization and gene duplications and losses can be counted when polyploidization is present. Importantly, GRAMPA is also useful in identifying ancient allopolyploidization, which is otherwise difficult to detect due to the complexity of diploidization (Gregg et al., 2017).

In this study, based on the high-quality whole genome data collected from 24 species representing six main clades of Superasterids, we revealed the allopolyploid origin of Ericales with a gene-tree reconciliation algorithm and investigated the spectrum of gene loss/retention that followed. Our study is important for exploring ancient allopolyploidization in Ericales, gene loss and functional divergence after WGD, and its implications for genetic variation and adaptive phenotypic diversity in Ericales. It also provides perspectives on the role of ancient hybridization in surviving global climate changes.

## Materials and methods

### Data collection

To resolve a backbone phylogeny of Superasterids, especially of Ericales, the genomic sequences and annotations were firstly collected from ~200 published Superasterids genomes (https://plabipd.de/plant_genomes_pa.ep). Then, we applied three selective criteria to the collected genomic data, 1) the coding gene sequence (cds) and generic feature format (gff) files, and genome data needed to be accessible, 2) they should cover the major taxa of Superasterids as much as possible, and 3) are assembled and annotated with higher completeness and continuity. Finally, we chose 24 genomes/species: seven from Lamiids (*Antirrhinum majus*, *Salvia miltiorrhiza*, *Osmanthus fragrans*, *Ophiorrhiza pumila*, *Coffea humblotiana*, *Ipomoea triloba*, and *Solanum tuberosum*); three from Campanulids (*Lactuca sativa*, *Apium graveolens*, and *Lonicera japonica*) three Cornales (*Nyssa sinensis*, *Camptotheca acuminata*, and *Davidia involucrata*); three from Caryophyllales (*Atriplex hortensis*, *Simmondsia chinensis*, and *Fagopyrum tataricum*); one from Santalales (*Malania oleifera*, as outgroup); and seven from Ericales. Among the latter, the genomic dataset contained the main ecologically important lineages from tropical rainforests (*e.g*., Sapotaceae (*Diospyros oleifera*) and Ebenaceae (*Vitellaria paradoxa*)), heathlands (*e.g*., Ericaceae (*Rhododendron simsii* and *Vaccinium macrocarpon*)), open habitats (*e.g*., Primulaceae (*Aegiceras corniculatum*)), and other important ecosystems (Actinidiaceae (*Actinidia chinensis*) and Theaceae (*Camellia sinensis*)). Detailed sampling information can be found in **Supplementary Table 1**.

Gene functional annotations were performed using eggNOG-mapper v2 (Cantalapiedra et al., 2021) with default parameters. Higher proportions of genes (78-98%) were annotated for seven Ericales species (**Supplementary Table 8**).

### Phylogenetic inference

We first built a dataset by selecting only the longest transcript isoform of each gene. Further, the protein sequences were grouped into orthogroups using Orthofinder (v 2.5.2) (Emms and Kelly, 2019) with the following parameters: -M msa -S diamond -A mafft -T fasttree. We extracted 34 single-copy and 532 low-copy orthogroups (with a minimum of 80% of the species having single-copy genes in any orthogroup) for phylogenomic analyses. The protein sequences from each orthogroup were aligned using MAFFT v7.407 (Katoh and Standley, 2013), and the amino acid alignments were then converted into codon-preserving alignments using PAL2NAL v.14 (Suyama et al., 2006). Finally, two strategies (concatenation and coalescent-based) were used for phylogenetic analyses with different alignments for single- and low- copy orthogroups, respectively. For the concatenation-based analyses, gene alignments were concatenated as a single supermatrix and the tree was inferred by IQ-TREE v2.1.2 (Nguyen et al., 2015) with automatic selection of the best-fit substitution model (-m MFP) and 1000 ultrafast bootstrap replicates (-bb 1000). For coalescent-based analyses, individual trees were constructed with amino acid alignments by ultrafast bootstrap with 1000 replicates. Then, the maximum likelihood (ML) gene trees with a bootstrap support cutoff value of 50% were used to construct species trees *via* ASTRAL-pro v1.1.2 (Zhang et al., 2020a) for low copy orthogroups. We also used single-copy orthogroups to generate a species tree with ASTRAL v5.7.8 (Zhang et al., 2018).

### Inference of allopolyploidization

GRAMPA (Gregg et al., 2017) was employed to infer the potential allopolyploid origin of Ericales with three strategies of species sampling. In the first strategy, seven representative Ericales plants were selected including four species from core Ericales (Ericaceae (*R. simsii* and *V. macrocarpon*, Actinidiaceae (*A. chinensis*), and Theaceae (*C. sinensis*)), and three species from other Ericales (Sapotaceae (*D. oleifera*), Ebenaceae (*V. paradoxa*), and Primulaceae (*A. corniculatum*)). In the second strategy, a total of 16 sampling designs were performed by randomly subsampling Ericales species (from two to six) to investigate the impact of taxon sampling. In the third strategy, we investigated five designs to confirm whether clades of Asterids (Lamiids, Campanulids, and Cornales) could have contributed parental lineages of the WGD event within Ericales. *Malania oleifera* was used as an outgroup in each sampling strategy.

For each taxon sampling strategy, GRAMPA was used to resolve WGD events, and the input files were a species tree and a set of gene trees. Furthermore, GRAMPA returns cases of gene duplication and loss, and a total reconciliation score (sum of duplication and loss) for species tree and individual gene tree. Species trees were pruned using Newick Utilities for each sampling. Rooted gene trees were generated by Orthofinder based on different species samplings with *M. oleifera* as outgroup. The preliminarily filtered gene trees were obtained by removing the trees with more than eight polyploid groups per single gene tree, and this computation was implemented with GRAMPA. To avoid overcounting reconciliation scores, deep filtered gene trees were generated from preliminarily filtered gene trees by controlling the gene copy number, which is allowable to be more than one and less than four per species.

The gene trees were classified to different gene retention/loss scenarios (T1: (OtherEricales+, (OtherEricales*, CoreEricales)), T2: ((OtherEricales, CoreEricales+), CoreEricales*), and T3: ((OtherEricales*, CoreEricales+), (OtherEricales+, CoreEricales*))) with Newick Utilities. COG functional enrichment analysis was conducted for each orthogroup following different gene retention/loss scenarios (T1, T2 and T3 topologies, see above) (**Figures 1B, C**) with the program enricher in R package clusterProfiler (Yu et al., 2012), and the *p* values were adjusted using the Benjamini–Hochberg procedure.

## Results and discussion

### Conflicts in phylogenetic inferences

Here we collected high-quality whole genome data of 24 species representing the six main clades of Superasterids (**Figure 1A** and **Supplementary Table 1**). Benchmarking Universal Single Copy Orthologs (BUSCO) analysis (Simão et al., 2015) supported the high completeness in both the predicted gene sets (73 - 99% of complete genes) (**Figure 1B** and **Supplementary Table 2**), and assemblies (90 - 98%) (**Figure 1C**). Additionally, most genome assemblies are already at the pseudochromosome level with high contig N50 (**Figure 1D** and **Supplementary Table 3**). This highly accurate and complete dataset allowed for the confident phylogenomics in Superasterids, especially Ericales.

**Figure 2:**
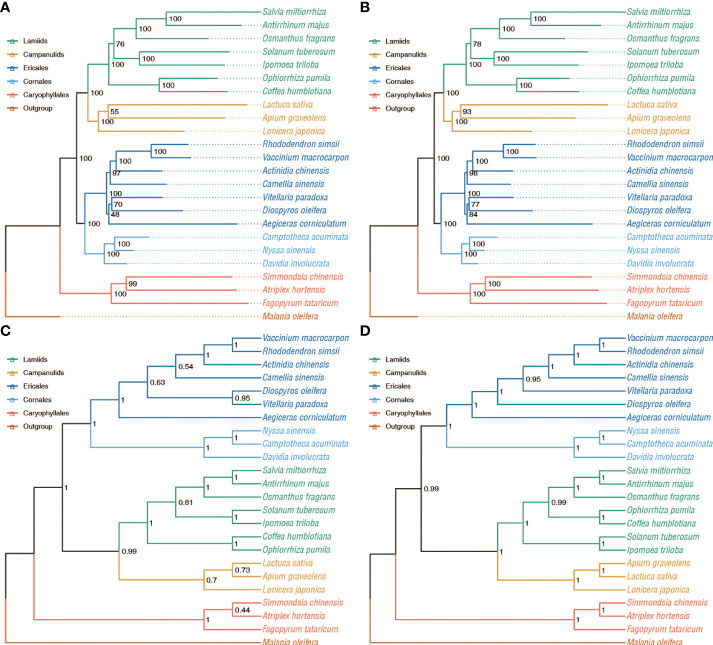
Phylogenetic relationships inferred from the 34 single-copy orthogroups and 532 low-copy orthogroups with concatenation and coalescent-based approaches. Bootstrap support (BS) values are shown. **(A)** Concatenation-based inference using the CDS sequence alignment from 34 single-copy orthogroups. **(B)** Concatenat-based inference using the amino acid sequence alignment from 34 single-copy orthogroups. **(C)** Coalescent-based inference using the amino acid sequence alignment from 34 single-copy orthogroups. **(D)** Coalescent-based inference using the amino acid sequence alignment from 532 low-copy orthogroups with Bootstrap support (BS) values ≥ 50%.

We obtained 34 single-copy and 532 low-copy orthogroups (**Supplementary Table 4**), and these two data sets were used to reconstruct concatenation and coalescent-based phylogenetic trees (**Figures 1A**, **2A–D**) using nucleotide and amino acid sequences, respectively. The five maximum likelihood (ML) trees well resolved the relationships among the six main clades of Superasterids, such as Lamiids and Campanulids as sister lineages and forming the clade Gentianidae. And Cornales and Ericales are of reciprocal monophyly, forming a sister clade to the rest of the Asterids. This topology agrees with the nuclear gene-based phylogenetic tree (Stull et al., 2020; Zhang et al., 2020b; Zhao et al., 2021; Baker et al., 2022), but conflicts with plastid phylogenies (APG IV, 2016; Li et al., 2019), in which Cornales is found at the base of Asterids. The concatenation-based phylogenetic trees all supported core Ericales and other Ericales as sister lineages (**Figures 1A**, **2A, B**), which is consistent with published reports (Schönenberger et al., 2005; APG IV, 2016; Larson et al., 2020; Baker et al., 2022) using genomic and transcriptomic data with nuclear or/and plastid sequences from expanded sampling. The relationships within Ericales were conflicting between the concatenated (**Figures 1A**, **2A, B**) and the coalescent phylogenies (**Figures 2C, D**). On the coalescent phylogenies some relationships within Ericales remain unresolved, such as the relationships among Ebenaceae, Primulaceae, and Sapotaceae. The conflicts within Ericales are presumably attributable to hybridization and WGD (Larson et al., 2020; Stull et al., 2020; Zhang et al., 2020b; Wang et al., 2021). To accommodate the among-gene heterogeneity in the evolutionary process and investigate possible ancient WGDs, we used the concatenation tree consistent with previous representations (Schönenberger et al., 2005; APG IV, 2016; Larson et al., 2020; Baker et al., 2022) as a reference in the following analyses.

### Potential allopolyploid origin of Ericales

**Figure 3:**
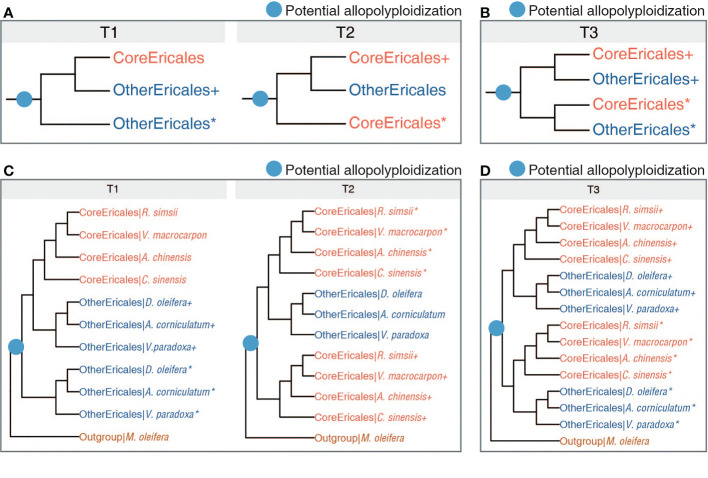
Evolutionary history. **(A)** Two possible multiple-labelled (MUL) topologies following the identified allopolyploidization (blue dot). T1 and T2 represent the scenarios with one copy of duplicated genes loss following the identified allopolyploidization. **(B)**. One possible MUL topology (T3) indicating the scenario where two duplicated gene copies were retained following the identified allopolyploidization. **(C, D)** The referenced multi-labelled species trees supporting T1, T2 and T3, respectively. “*” and “+” respectively denote two copies of a gene duplication generated from one WGD, or respectively represent two descendants of different parental lineages in a hybrid genome.

We used an LCA-based algorithm, implemented in GRAMPA, to resolve potential allopolyploidization and the parental lineages, and to investigate gene duplication and loss in each individual gene tree by matching gene trees with the species tree. In the GRAMPA analysis, lower reconciliation scores indicate that gene trees are more congruent with species tree, and that the species tree therefore better reflects the congruence among gene trees. We first reconstructed 15,143 gene trees with a preliminarily filtered dataset that incorporated seven Ericales plants (**Supplementary Table 5**). To avoid overcounting reconciliation scores, a total of 6,531 gene trees were further generated from the deep filtered dataset, ensuring that the gene number ranged between one and five per species (**Supplementary Table 5**). We obtained 21,674 gene trees from the datasets of two filtering modes, which we matched to 181 possible species trees (**Supplementary Table 5**). All the top ten species trees with the lowest total reconciliation score (**Supplementary Figures 1**, **2**) were concordant with one of the two multi-labelled (MUL) topologies [T1: (OtherEricales+, (OtherEricales*, CoreEricales)] and T2: [(OtherEricales, CoreEricales+), CoreEricales*)] (**Figure 3A**). To examine the influence of limited taxa sampling, we obtained 259,774 gene trees based on the datasets generated from 16 subsamplings of Ericales species (**Supplementary Table 6**). All 16 MUL trees with the lowest total reconciliation score were congruent with one of these two topologies, T1 and T2 (**Supplementary Figure 3**). These two topologies, supported by massive gene trees, were generated from different sampling and subsampling strategies. Topologies T1 and T2 were revealing lineage-specific gene loss following a potential allopolyploid origin of Ericales {T3: [(OtherEricales*, CoreEricales+), (OtherEricales+, CoreEricales*)]} (**Figure 3B**), while rejecting WGD events at the common ancestor of the core Ericales or at the common ancestor of Ericaceae and Actinidiaceae. In equation of each potential MUL topology (here, T1 to T3), “*” and “+” respectively denote two copies of a gene duplication generated from one WGD, or respectively represent two descendants of different parental lineages in a hybrid genome (whatever it is a homoploid or a polyploidy hybrid).

In the present study, we focused on a potentially ancient polyploid origin of the order Ericales. The topology T3 is for the scenario of potential allopolyploid origin of Ericales without lineage-specific gene loss, while T1 and T2 are for scenarios resulted from lineage-specific gene loss following an allopolyploidization, which was well supported here. We cannot rule out that a recent autopolyploidization (where interspecific divergence is shallow) may also result in gene family showing T1 or T2 topologies. But it is not possible that an ancient autopolyploidization may give T1 or T2 topologies, as in this case all these two are requiring deeply diverged gene copies from different lineages (parental genomes of an allopolyploid).

Cornales was previously considered as one of the possible parental lineages that hybridized with Gentianidae to form Ericales, as shown by phylogenetic network analyses (Stull et al., 2020). If Cornales and Gentianidae were parental lineages of the potential allopolyploid, the topology [(Cornales, Ericales+), (Gentianidae, Ericales*)] should be supported by numerous gene trees. However, the putative topology was not supported by the 15,116 to 16,307 filtered gene trees generated from the five subsampling designs (**Supplementary Figure 4** and **Supplementary Table 7**), and hence did not support Cornales and Gentianidae (Lamiids and Campanulids) as parental lineages of Ericales.

Our present study is formed up based on these unresolved questions, especially the potential ancient WGD and hybridization origin of Ericales. Different from routinely performed methods in inference of hybridization and WGD (synteny analysis and karyotype examination, or Ks-based, least common ancestor (LCA) reconciliation, gene networks, and count-based phylogenetic computation) (Schönenberger et al., 2005; Shi et al., 2010; Landis et al., 2018; Wei et al., 2018; Leebens-Mack et al., 2019; Chen et al., 2020; Larson et al., 2020; Stull et al., 2020), we for the first time discovered the evidence supporting the potential allopolyploid origin of Ericales, with a gene-tree reconciliation strategy. As one of the ancient WGD events during the Cretaceous, the allopolyploidy event could have contributed to the adaptation and radiation of Ericales under the drastic climate change in the Cretaceous (Fawcett et al., 2009; Zhang et al., 2020c).

### Gene loss after allopolyploidization

**Figure 4:**
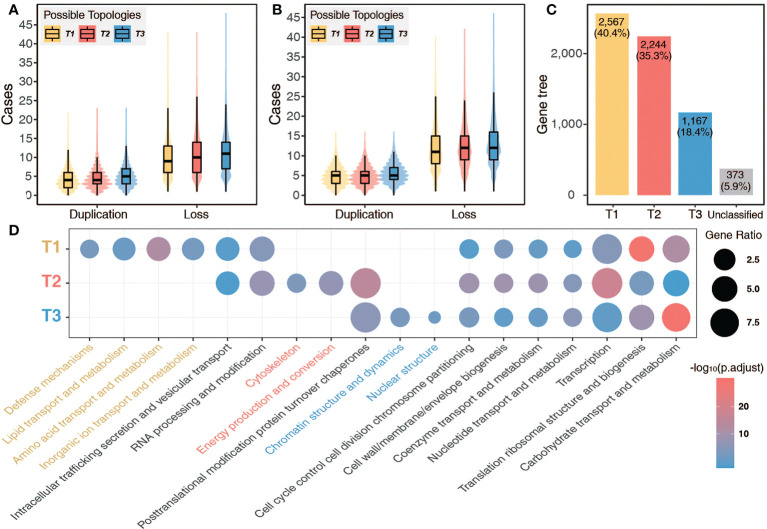
Gene loss after allopolyploidization. **(A, B)** Cases of gene duplication and loss in each gene tree by matching preliminarily filtered **(A)** and deep filtered **(B)** gene trees with three possible topologies, respectively. **(C)** Number of gene trees supporting the three possible MUL tree topologies. **(D)** Clusters of Orthologous Groups (COG) functional enrichment of orthogroups following the different gene retention/loss scenarios (T1, T2, and T3).

To analyze the extent of gene loss and retention following the ancient allopolyploidization of Ericales, we reconstructed the three MUL trees (simplified as T1, T2, and T3) as reference (**Figures 3A–D**) and further counted the cases of gene loss and retention revealed in each gene tree. The 21,674 gene trees generated from the preliminarily filtered and deep filtered datasets were aligned with the three MUL trees. We found that more than 95% of the gene trees showed more gene losses than retentions in the deep filtered dataset, and more than 90% in the preliminarily filtered dataset (**Figures 4A, B** and **Supplementary Table 5**), suggesting widespread gene loss following allopolyploidization (**Figures 4A, B**). Therefore, the question arises whether the gene losses after allopolyploidization are lineage-specific or random. We detected 2,567 (40.4%) gene trees following T1 topology and 2,244 (35.3%) cases for T2, which showed genome-wide pattern of lineage-specific gene loss/retention of one copy in core Ericales and other Ericales, respectively. Besides, 1,167 (18.4%) gene trees were congruent with T3 topology, indicating a small proportion of gene pairs retained after allopolyploidization, and a few (corresponding to only 5.9%) unclassified cases (**Figure 4C**). This large number of reciprocal loss of gene pairs, as well as the competing relationships among T1 and T2 topologies, might be the key factors that resulted in the difficulty to identify homologous relationships among genes, and the problem in reconstructing phylogenetic relationships and tracing the evolutionary origin of Ericales (Yang and Rannala, 2012; Stull et al., 2020; Xiong et al., 2022).

Genes following the T1 topology were biased toward distinct Clusters of Orthologous Groups (COG) categories related to defense mechanisms, lipid transport and metabolism, amino acid transport and metabolism, inorganic ion transport and metabolism. Genes following T2 topology were biased toward COG categories related to cytoskeleton, energy production and conversion (**Figure 4D**). Functional enrichment to basic biological functions, such as transcription, translation, ribosomal structure and biogenesis, and carbohydrate transport and metabolism, were shared among cases following all three MUL tree topological scenarios (T1, T2 and T3) (**Figure 4D**). Gene loss following WGD is often biased (Albalat and Cañestro, 2016; Mandakova and Lysak, 2018; Xiong et al., 2022), with one subgenome retaining more ancestral genes and the other sustaining more gene deletions, which may lead to the functional divergence between subgenomes and gene pairs after WGD (Liang and Schnable, 2018).

Our results reveal the allopolyploid origin of Ericales, while neither Cornales, Campanulids, nor Lamiids were supported to be the parental lineages. Extensive lineage specific gene loss and biased gene retention following polyploidization were evidenced, which may be associated with functional divergence and cause adaptive phenotypic diversity in Ericales. Such an ancient allopolyploid origin of a species-rich lineage could provide phylogenomically conflicting signals in attempts to fully infer phylogenetic relationship and evolutionary history. Newly formed allopolyploids include the widely grown crops wheat, cotton, and canola, but relatively little is known about ancient allopolyploidization. It is therefore of great value that more whole-genome data will be available in the future to further explore the evolutionary history of this ancient allopolyploidy event. Here, we also presented a research idea that we believe will spark further discussion as more taxa are sampled and more gene functions are discovered in the future.

## Data availability statement

The original contributions presented in the study are included in the article/**Supplementary Material**. Further inquiries can be directed to the corresponding authors.

## Author contributions

F-SY and J-FM conceived and designed the study. SN, X-CT, LK, S-WZ, Z-YC, J-SQ and WZ prepared the materials, conducted the experiments, analyzed data and prepared results. J-FM, F-SY and SN wrote the manuscript. Y.A.E.K. and I.P. were involved in finalizing the manuscript draft. All authors read and approved the final draft. All authors contributed to the article and approved the submitted version.

## Funding

This study was supported by National Natural Science Foundation of China (32171816). This work was conducted in part during a study visit by the first author to the Department of Forest and Conservation Sciences at the University of British Columbia supported by China Scholarship Council.

## Conflict of interest

The authors declare that the research was conducted in the absence of any commercial or financial relationships that could be construed as a potential conflict of interest.

## Publisher’s note

All claims expressed in this article are solely those of the authors and do not necessarily represent those of their affiliated organizations, or those of the publisher, the editors and the reviewers. Any product that may be evaluated in this article, or claim that may be made by its manufacturer, is not guaranteed or endorsed by the publisher.
