# Supplementary material for: Potential allopolyploid origin of Ericales revealed with gene-tree reconciliation
Source: Front Plant Sci. 2022 Nov 15;13:1006904. doi: 10.3389/fpls.2022.1006904 (PMC9706204; doi:10.3389/fpls.2022.1006904)
Supplement: Supplementary file 1 [file DataSheet_1.pdf]

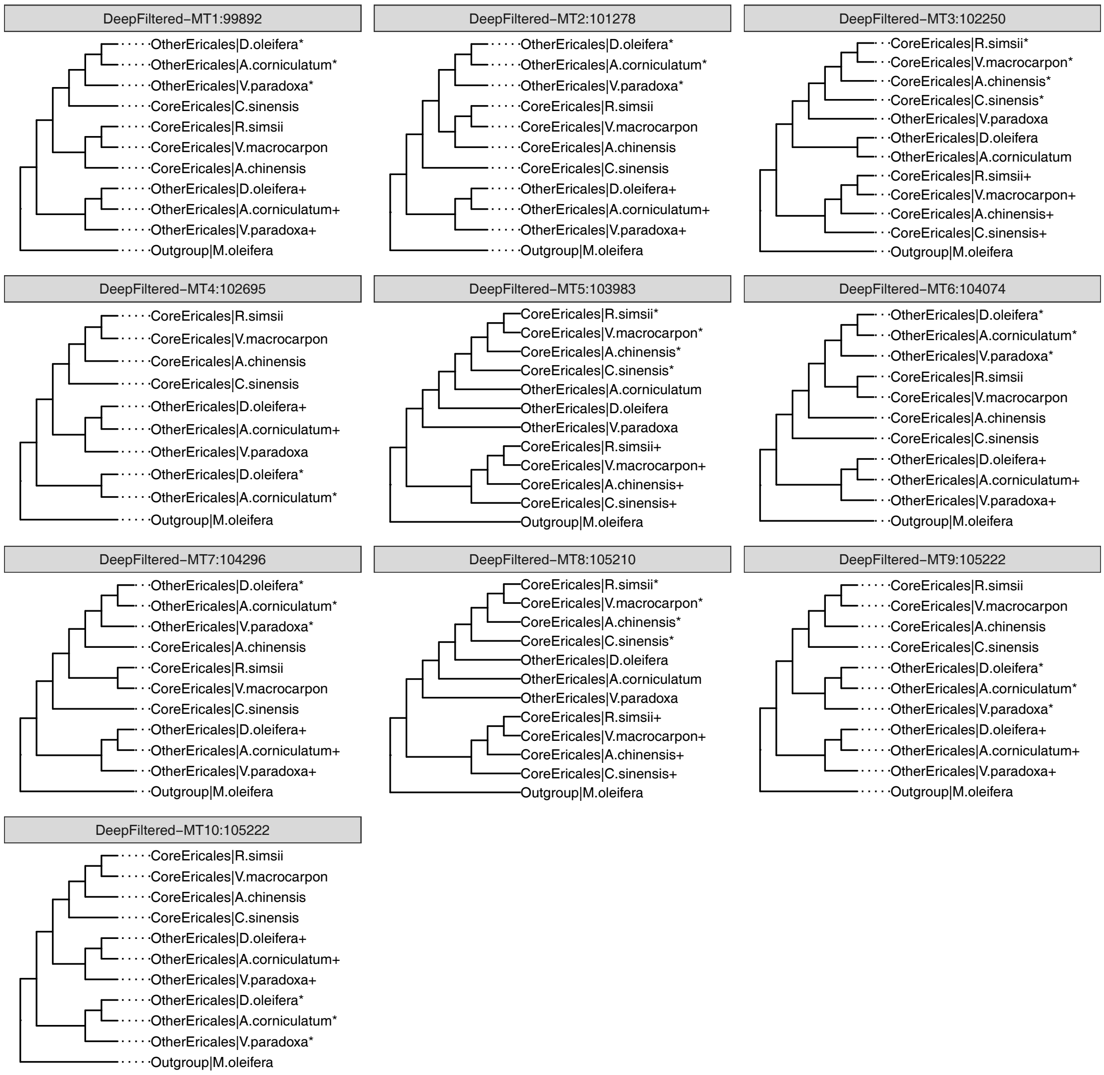

Supplementary Figure 1: Top 10 multi-labelled species trees (MT) with the lowest total reconciliation score by matching preliminarily filtered gene trees with the reference species tree. Ericales (Core Ericales+other Ericales). "*" and "+" respectively denote two copies of a gene duplication generated from one WGD, or respectively represent two descendants of different parental lineages in a hybrid genome (whatever it is a homoploid or a polyploidy hybrid).

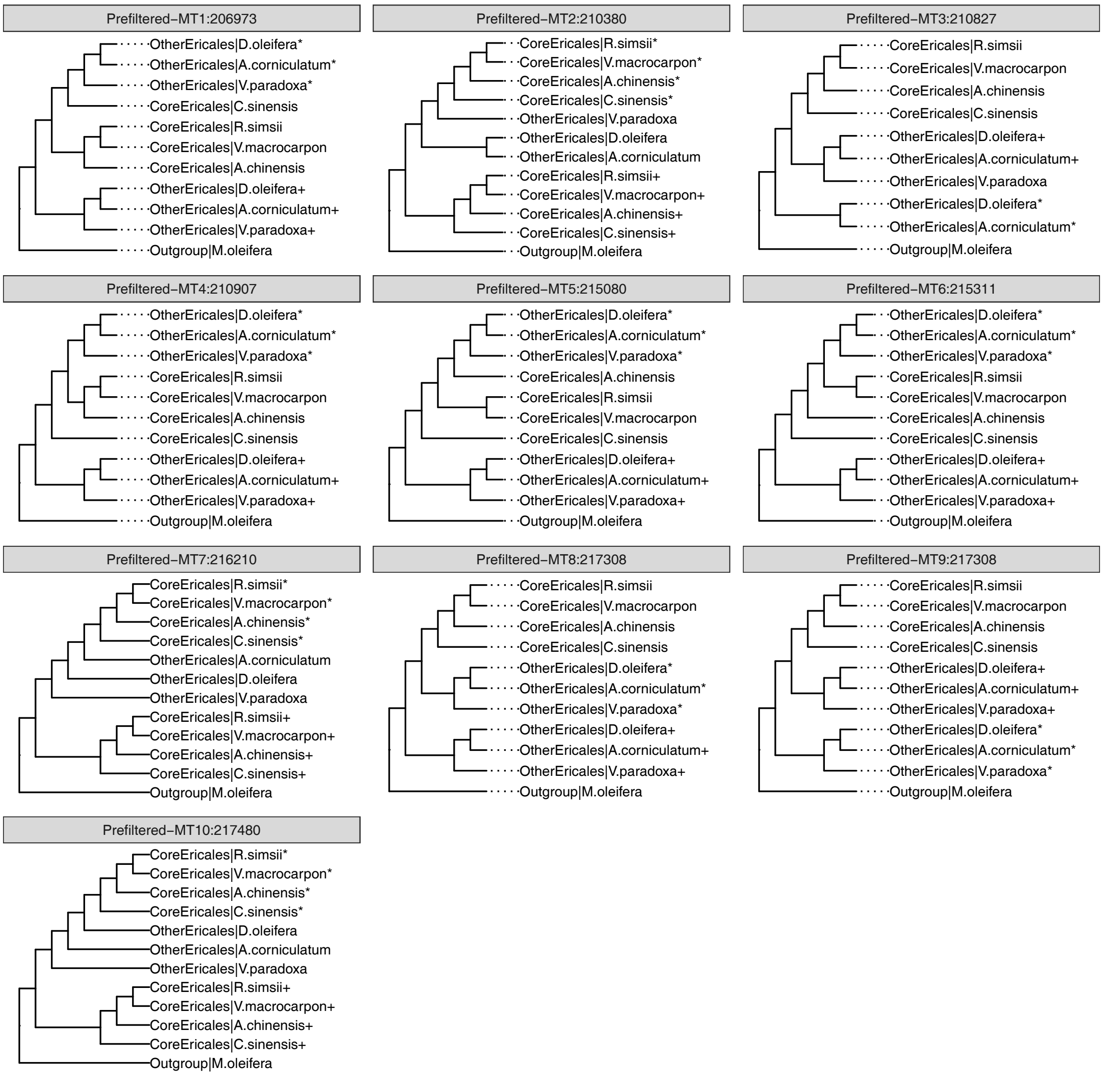

Supplementary Figure 2: Top 10 multi-labelled species trees (MT) with the lowest total reconciliation score by matching deep filtered gene trees with the reference species tree. Ericales (Core Ericales+other Ericales). "*" and "+" respectively denote two copies of a gene duplication generated from one WGD, or respectively represent two descendants of different parental lineages in a hybrid genome (whatever it is a homoploid or a polyploidy hybrid).

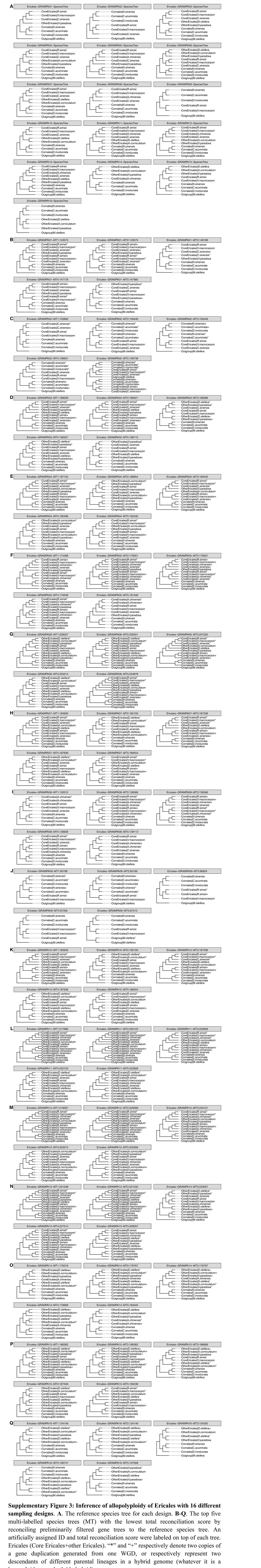

Supplementary Figure 3: Inference of allopolyploidy of Ericales with 16 different sampling designs. **A**. The reference species tree for each design. **B-Q**. The top five multi-labelled species trees (MT) with the lowest total reconciliation score by reconciling preliminarily filtered gene trees to the reference species tree. An artificially assigned ID and total reconciliation score were labeled on top of each tree. Ericales (Core Ericales+other Ericales). "*" and "+" respectively denote two copies of a gene duplication generated from one WGD, or respectively represent two descendants of different parental lineages in a hybrid genome (whatever it is a homoploid or a polyploidy hybrid).

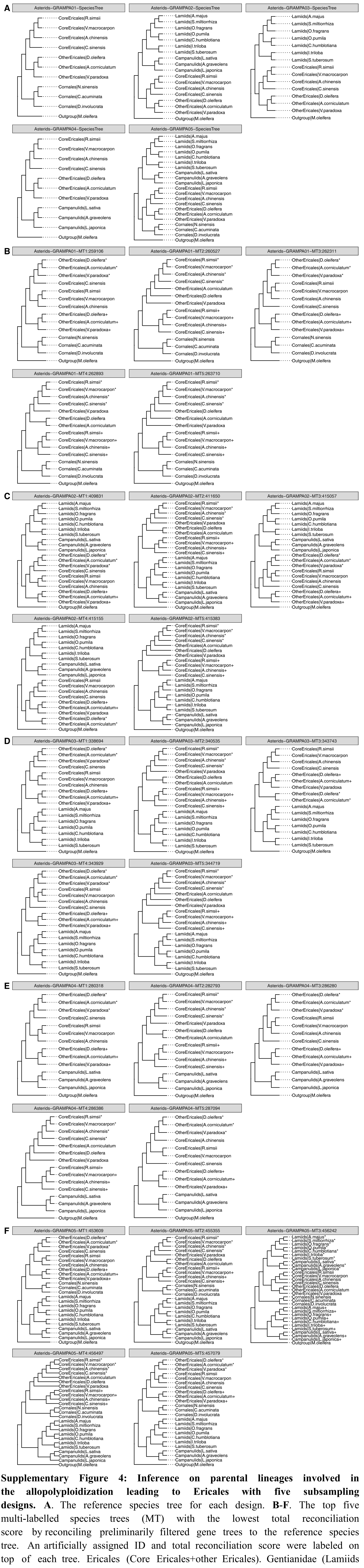

Supplementary Figure 4: Inference on parental lineages involved in the allopolyploidization leading to Ericales with five subsampling designs. A. The reference species tree for each design. **B**-**F**. The top five multi-labelled species trees (MT) with the lowest total reconciliation score by reconciling preliminarily filtered gene trees to the reference species tree. An artificially assigned ID and total reconciliation score were labeled on top of each tree. Ericales (Core Ericales+other Ericales). Gentianidae (Lamiids +Campanulids). "*" and "+" respectively denote two copies of a gene duplication generated from one WGD, or respectively represent two descendants of different parental lineages in a hybrid genome (whatever it is a homoploid or a polyploidy hybrid).

Supplementary Table 1: Statistics of the phylogeny for 24 species from Superasterids.

Species	Family	Order	DOI
Apium graveolens	Apiaceae	Apiales	doi.org/10.1111/pbi.13499
Lactuca sativa	Asteraceae	Asterales	doi.org/10.1038/ncomms14953
Atriplex hortensis	Amaranthaceae	Caryophyllales	doi.org/10.3389/fpls.2020.00624
Fagopyrum tataricum	Polygonaceae	Caryophyllales	doi.org/10.1016/j.molp.2017.08.013
Simmondsia chinensis	Simmondsiaceae	Caryophyllales	doi.org/10.1126/sciadv.aay3240
Camptotheca acuminata	Nyssaceae	Cornales	doi.org/10.1038/s41467-021-23872-9
Davidia involucrata	Nyssaceae	Cornales	doi.org/10.1111/1755-0998.13138
Nyssa sinensis	Nyssaceae	Cornales	doi.org/10.1038/s41597-019-0296-y
Lonicera japonica	Caprifoliaceae	Dipsacales	doi.org/10.1111/nph.16552
Actinidia chinensis	Actinidiaceae	Ericales	doi.org/10.1186/s12864-018-4656-3
Diospyros oleifera	Ebenaceae	Ericales	doi.org/10.1093/gigascience/giz164
Rhododendron simsii	Ericaceae	Ericales	doi.org/10.1038/s41467-020-18771-4
Vaccinium macrocarpon	Ericaceae	Ericales	doi.org/10.3389/fpls.2021.633310
Aegiceras corniculatum	Primulaceae	Ericales	doi.org/10.1111/1755-0998.13347
Vitellaria paradoxa	Sapotaceae	Ericales	doi.org/10.3389/fpls.2021.720670
Camellia sinensis	Theaceae	Ericales	doi.org/10.1038/s41438-020-0288-2
Coffea humblotiana	Rubiaceae	Gentianales	doi.org/10.1038/s41598-021-87419-0
Ophiorrhiza pumila	Rubiaceae	Gentianales	doi.org/10.1038/s41467-020-20508-2
Salvia miltiorrhiza	Lamiaceae	Lamiales	doi.org/10.1186/s13742-015-0104-3
Osmanthus fragrans	Oleaceae	Lamiales	doi.org/10.1038/s41438-018-0108-0
Antirrhinum majus	Plantaginaceae	Lamiales	doi.org/10.1038/s41477-018-0349-9
Malania oleifera	Olacaceae	Santalales	doi.org/10.1093/gigascience/giy164
Ipomoea triloba	Convolvulaceae	Solanales	doi.org/10.1038/s41467-018-06983-8
Solanum tuberosum	Solanaceae	Solanales	doi.org/10.1534/g3.120.401550

Supplementary Table 2: Statistics of the coding genes for 24 species from Superasterids.

Species	Sequence NO.	Average Length (bp)	Complete BUSCOs
Apium graveolens	31,326	378.1	93.8
Lactuca sativa	38,910	372.6	98.1
Atriplex hortensis	36,985	326.3	86.8
Fagopyrum tataricum	31,839	336.9	89.4
Simmondsia chinensis	23,490	378.5	91
Camptotheca acuminata	27,940	432.7	93.2
Davidia involucrata	42,554	369	90.2
Nyssa sinensis	36,241	393.4	87.6
Lonicera japonica	33,939	363.4	82.1
Actinidia chinensis	33,044	425	94.9
Diospyros oleifera	30,530	360.2	85.4
Rhododendron simsii	32,999	428.6	90.3
Vaccinium macrocarpon	22,836	445	83.1
Aegiceras corniculatum	40,727	400.3	89.6
Vitellaria paradoxa	38,505	328	84.6
Camellia sinensis	32,770	444.3	74.7
Coffea humblotiana	32,874	333.5	77.3
Ophiorrhiza pumila	32,374	362.4	89.2
Salvia miltiorrhiza	29,236	408.5	97.2
Osmanthus fragrans	45,542	379.9	94.5
Antirrhinum majus	37,234	345.1	94.4
Malania oleifera	24,094	426	73.8
Ipomoea triloba	30,559	456.1	99.3
Solanum tuberosum	32,917	391.5	92.9

Supplementary Table 3: Statistics of the genome assemblies for 24 species from Superasterids.

Species	Complete BUSCOs	GC content	Contigs NO.	Contig N50 (bp)	Scaffolds NO.	Scaffold N50 (bp)
Apium graveolens	97.8	0.36	9,496	790,578	4,863	289,786,985
Lactuca sativa	98.4	0.38	361,797	12,459	10	257,910,700
Atriplex hortensis	97.5	0.37	3,620	816,576	1,325	98,884,393
Fagopyrum tataricum	94.9	0.39	9,476	423,036	7,020	53,883,329
Simmondsia chinensis	95.7	0.37	994	5,207,162	27	38,943,102
Camptotheca acuminata	96.5	0.33	1,130	1,473,707	775	18,276,129
Davidia involucrata	96.3	0.38	4,306	576,568	1,015	49,920,885
Nyssa sinensis	96	0.36	654	3,599,157	294	43,339,305
Lonicera japonica	97	0.34	920	2,148,893	145	84,431,753
Actinidia chinensis	96.7	0.35	26,755	34,807	1,234	18,944,233
Diospyros oleifera	95	0.37	3,022	2,851,023	2,533	45,442,152
Rhododendron simsii	96.8	0.39	911	2,234,511	552	36,350,743
Vaccinium macrocarpon	93.4	0.38	810	1,359,385	350	37,842,272
Aegiceras corniculatum	95.3	0.34	4,958	1,176,000	393	37,736,876
Vitellaria paradoxa	95.7	0.33	594	2,390,495	111	55,380,075
Camellia sinensis	94	0.38	116,346	58,407	14,412	218,115,851
Coffea humblotiana	90.4	0.36	804	1,451,456	390	29,629,744
Ophiorrhiza pumila	97.9	0.34	36	18,486,819	13	40,565,736
Salvia miltiorrhiza	97.3	0.38	1,487	2,705,653	982	69,825,357
Osmanthus fragrans	96.8	0.34	768	1,595,720	50	31,689,246
Antirrhinum majus	98.2	0.36	1,240	748,444	8	62,720,994
Malania oleifera	94.1	0.36	2,987	1,218,690	1,277	4,647,296
Ipomoea triloba	98.2	0.36	31,273	36,938	17	29,809,665
Solanum tuberosum	98	0.35	465	15,195,955	288	59,670,755

Supplementary Table 4: Statistics of gene family analyses for phylogenetic inferences.

	**Statistics**
Number of species	24
Number of genes	799,465
Number of genes in orthogroups	741,847
Number of unassigned genes	57,618
Percentage of genes in orthogroups	93
Percentage of unassigned genes	7
Number of orthogroups	36,551
Number of species-specific orthogroups	14,490
Number of genes in species-specific orthogroups	74,227
Percentage of genes in species-specific orthogroups	9
Mean orthogroup size	20
Median orthogroup size	6
G50 (assigned genes)	43
G50 (all genes)	40
O50 (assigned genes)	4,616
O50 (all genes)	5,315
Number of orthogroups with all species present	5,622
Number of single-copy orthogroups	34
Number of low-copy orthogroups	532

Supplementary Table 5: Statistics of reconciliation analysis.

Possible Topologies	Data Sets	Duplication	Loss	Reconciliation Scores	Gene Tree NO.	Gene Tree (Loss>Duplication) NO.
T1	Prefiltered	65,665	151,643	217,308	15,143	13,703 (90.49%)
T1	DeepFiltered	30,866	74,356	105,222	6,351	6,053 (95.31%)
T2	Prefiltered	67,526	160,578	228,104	15,143	13,791 (91.07%)
T2	DeepFiltered	31,495	78,349	109,844	6,351	6,118 (96.33%)
T3	Prefiltered	75,643	170,664	246,307	15,143	14,062 (92.86%)
T3	DeepFiltered	35,930	83,075	119,005	6,351	6,144 (96.74%)

Supplementary Table 6: Summary of 16 sampling designs within Ericales.

Sampling Designs	Gene Tree NO.	Gene NO.
Ericales-GRAMPA01	16,554	172,430
Ericales-GRAMPA02	16,256	151,720
Ericales-GRAMPA03	16,526	188,958
Ericales-GRAMPA04	16,299	185,572
Ericales-GRAMPA05	16,484	193,365
Ericales-GRAMPA06	16,101	196,044
Ericales-GRAMPA07	16,163	184,553
Ericales-GRAMPA08	16,351	176,986
Ericales-GRAMPA09	15,848	128,285
Ericales-GRAMPA10	16,163	184,553
Ericales-GRAMPA11	16,024	202,269
Ericales-GRAMPA12	16,113	201,740
Ericales-GRAMPA13	16,339	207,035
Ericales-GRAMPA14	16,186	172,712
Ericales-GRAMPA15	16,180	181,588
Ericales-GRAMPA16	16,187	151,542
Total	259,774	2,879,352

Supplementary Table 7: Summary of five subsampling designs within Asterids.

Sampling Designs	Gene Tree NO.	Gene NO.
Asterids-GRAMPA01	15,868	210,315
Asterids-GRAMPA02	15,895	282,523
Asterids-GRAMPA03	15,783	260,296
Asterids-GRAMPA04	15,116	194,000
Asterids-GRAMPA05	16,307	313,373
Total	78,969	1,260,507

Supplementary Table 8: Statistics of gene functional annotations for seven species from Ericales.

Species	COG	eggNOG	GO	KEGG	Annotated	Total
Actinidia chinensis	32,422	30,181	17,549	16,269	32,422 (98.12%)	33,044
Aegiceras corniculatum	32,181	30,524	15,807	15,652	32,181 (79.02%)	40,727
Camellia sinensis	31,725	29,962	16,772	16,066	31,725 (96.81%)	32,770
Diospyros oleifera	27,810	26,009	13,927	13,186	27,810 (91.09%)	30,530
Rhododendron simsii	29,574	27,732	14,204	13,727	29,574 (89.62%)	32,999
Vaccinium macrocarpon	22,034	20,939	12,087	11,869	22,034 (96.49%)	22,836
Vitellaria paradoxa	30,033	27,952	15,943	14,641	30,033 (78.00%)	38,505
